# Electrowetting-on-Dielectric Based Economical Digital Microfluidic Chip on Flexible Substrate by Inkjet Printing

**DOI:** 10.3390/mi11121113

**Published:** 2020-12-16

**Authors:** He Wang, Liguo Chen

**Affiliations:** 1 Robotics and Microsystems Center, Soochow University, Soochow 215006, China; 20154029004@stu.suda.edu.cn; 2 School of Mechanical Engineering, Henan University of Engineering, Zhengzhou 451191, China

**Keywords:** digital microfluidics, electrowetting-on-dielectric, inkjet printing, flexible substrate, dielectric wrap film, hexagon electrode

## Abstract

In order to get rid of the dependence on expensive photolithography technology and related facilities, an economic and simple design and fabrication technology for digital microfluidics (DMF) is proposed. The electrodes pattern was generated by inkjet printing nanosilver conductive ink on the flexible Polyethylene terephthalate (PET) substrate with a 3D circuit board printer, food wrap film was attached to the electrode array to act as the dielectric layer and Teflon^®^ AF was sprayed to form a hydrophobic layer. The PET substrate and food wrap film are low cost and accessible to general users. The proposed flexible DMF chips can be reused for a long time by replacing the dielectric film coated with hydrophobic layer. The resolution and conductivity of silver traces and the contact angle and velocity of the droplets were evaluated to demonstrate that the proposed technology is comparable to the traditional DMF fabrication process. As far as the rapid prototyping of DMF is concerned, this technology has shown very attractive advantages in many aspects, such as fabrication cost, fabrication time, material selection and mass production capacity, without sacrificing the performance of DMF. The flexible DMF chips have successfully implemented basic droplet operations on a square and hexagon electrode array.

## 1. Introduction

The digital microfluidics system has developed rapidly in last few years [1] in response to the need for handling and manipulating pico-to-microliter volume of fluids [2,3]. It has great potential for point-of-care applications such as in the field of biochemical analysis, clinical diagnosis, environmental monitoring and food safety analysis in recent years. In digital microfluidics (DMF) devices, discrete droplets containing different biochemical samples or reagents are typically manipulated for various analysis and detection by applying a series of electrical potentials to an array of electrodes coated with a dielectric layer and a hydrophobic layer, which is referred to as electrowetting-on-dielectric (EWOD), one of the most common actuation mechanisms. DMF have the capacity to address each droplet individually [4]. It can effectively avoid the cross-contaminations, greatly reduce reagent and sample consumption and the time of biochemical reactions, and achieve precise quantitative control.

For the DMF chip, an array of patterned metal electrodes, the thin dielectric layer and the good hydrophobic layer must be processed. In the traditional fabrication technology, the patterned electrodes are fabricated by standard metal photolithography and sputtering on glass and silicon substrate, or etching on printed circuit boards based on copper substrate [5,6,7,8,9]. The former needs a clean-room facility while the latter adds to the complexity as it required additional chemical etching and the printed circuit board (PCB)-based DMF chip has the deep trenches between electrodes that maybe hinder the droplet movement [5]. The thin dielectric layer is deposited on the electrodes, which can be accomplished in different manners, depending on the dielectric material, such as vapor deposition of parylene or amorphous fluoropolymers, chemical vapor deposition of silicon nitride, spin coating of PDMS or SU-8, or thermal growth for silicon oxide [5,10,11,12]. The good hydrophobic layer is usually Teflon^®^ AF by spin coating, but these generation methods of the dielectric and hydrophobic layer usually also require a clean-room facility. It is impossible for general users to manipulate the micromachining facilities required to fabricate microfluidic chips. There are only two channels for most users to obtain DMF chips in general. Either participate in relevant training courses provided by a few professional companies or research institutions, pass the assessment, and then manipulate the facilities to fabricate the chips by yourself, or entrust them to be fabricated by someone else. However, regardless of the methods, the services provided by these companies or institutions can easily become too expensive because they charge for equipment usage, chip design and fabrication, etc. Due to high material and fabrication cost, high time consumption, lack of rapid prototyping, stringent requirements on the clean-room facility, and difficulty of optical detection, the traditional fabrication process of a digital microfluidic device is a bottleneck for the DMF accessibility in laboratories, which decrease its superiority and in particular are not well fit for point-of-care testing (POCT) for low-resource settings, such as remote areas and developing nations. The development of a low cost DMF chip, selection and utilization of the simple material deposition and improvement of the droplet operation and detection performance are major challenges for the existing fabrication process.

With the rapid development of printing technology, it has evolved from the office application to an important tool for industrial production. Printed electronics is widely gaining much attention and is increasingly used in flexible MEMS devices [13,14,15,16]. Therefore, the DMF chips on various flexible substrates via printing technology have recently emerged as a novel platform for simple, portable and low-cost DMF devices [17,18,19,20]. Two printing technologies, inkjet printing and screen printing, have been demonstrated to fabricate DMF Electrodes on flexible substrates [21,22,23,24,25,26,27]. In some reported literatures [21,22,23,24], although inkjet printing has replaced photolithography technology to process the electrode and got rid of the dependence on photolithography facilities and clean-rooms, high-cost dielectric material (parylene-C) and hydrophobic material (Teflon^®^ AF) are used. The coating of the two materials still needs to rely on expensive facilities and time-consuming process technologies such as chemical vapor deposition and spin coating, so it is still expensive from the perspective of cost. In addition, with such dielectric layer and hydrophobic layer coating technologies, once dielectric breakdown, poor hydrophobic effect or other chip failure occurs, the chip is very likely to be scrapped and cannot be reused, which will lead to the increase in the use cost of the chip. In other studies [25,26,27], electrodes have been fabricated by screen printing, and cheap dielectric and hydrophobic material (e.g., parafilm, transparent adhesive tape, rain repellent and SI 7200) are used. However, the original thickness of parafilm and transparent adhesive tape is 120 μm and 30 μm, and the initial contact angle without applied voltage is small. At such a high dielectric layer thickness and small contact angle, the droplet operation either sacrifices its movement velocity or requires a high voltage (300 V_rms_).

Following the DMF fabrication principle based on “out of clean-room”, we introduce a simple, convenient and low-cost way to fabricate a DMF chip which is barely restricted by the harsh fabrication environment as the traditional processing technology, especially suitable for household use or low-resource settings. Inkjet printing replaces photolithography to form electrodes, cheap food wrap film replaces expensive traditional dielectric materials to form dielectric layer, and then the hydrophobic layer is generated by spray-coating Teflon^®^ AF. Through the combination of the above three simplified material deposition methods, we successfully fabricated the economical flexible DMF chips (FDMFC).

## 2. Design, Materials and Fabrication Methods for FDMFC

### 2.1. Chip Design

**Figure 1:**
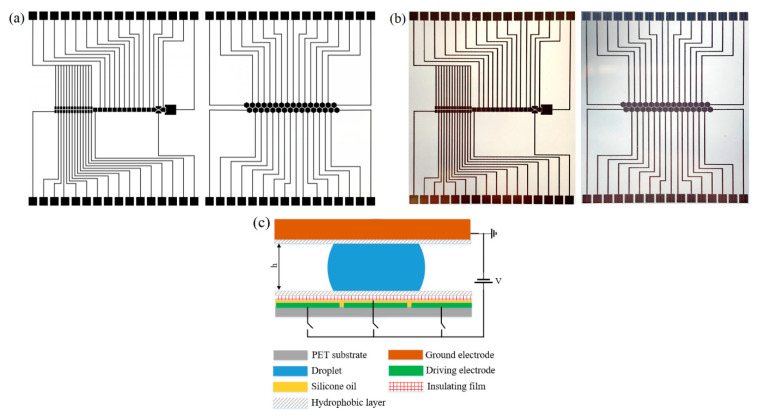
Chip design. (**a**) Photos of flexible DMF chips (FDMFC) structure diagram with design I and design II drawn by Microsoft Visio; (**b**) Photos of the chip printed on PET substrate corresponding to (a); (**c**) Side-view schematics of a FDMFC.

The FDMFC are shown in Figure 1. Two different chip structure diagrams are designed. Design I includes 1 reservoir electrodes (2.4 mm × 2.7 mm), 12 square driving electrodes (1 mm × 1 mm) and 4 dispensing electrodes with special shapes, and design II features 32 hexagon driving electrodes with a side length of 0.707 mm. The gap between adjacent electrodes and the width of traces connecting electrodes to contact pads in both designs are both 180 μm. The bottom plate is formed by inkjet-printing an array of patterned electrodes with nanosilver conductive ink on PET substrate. we chose household food wrap films attached to the electrodes as the dielectric layer to simply the deposition process; furthermore, although Teflon^®^ AF has been commonly used for the deposition of hydrophobic layer by spin coating, we tried a simpler and cheaper way to fabricate the hydrophobic layer, that is, to spray Teflon^®^ AF on the dielectric layer with a common spray bottle. The top plate is composed of an indium tin oxide (ITO) glass slide coated with Teflon^®^ AF. These simplified material deposition methods allow general users to easily fabricate a FDMFC, especially at home or in low-resource settings.

### 2.2. Materials

Nanosilver particle conductive ink and PET substrates are purchased from Beijing BroadTeko Intelligent Technology Co., Ltd. (Beijing, China). The particle size, viscosity and surface tension of the conductive ink are less than 50 nm, 5–20 cP and 27–33 dyn/cm, respectively. After the ink is printed, it needs to be sintered at 120° for 30 min. We selected a variety of food wrap films that are portable and easily accessible on the internet and in the supermarket as dielectric layers. We brought four brands of PVDC wrap films (Saran Wrap, Tokyo, Japan; Uncle Tom, Los Angeles, CA, USA; Miaojie, Wuxi, China; Youmiao, JiaXing, China) and PMP wrap film (Miaojie, Wuxi, China). The Teflon^®^ AF 1600 (Dupont, Wilmington, NC, USA) are used as the hydrophobic layer. Silicon oil with a kinematic viscosity of 10 cst (Dow Corning, Midland, MI, USA) acts as lubrication and medium filling in FDMFC.

### 2.3. Selection of Flexible Substrate

As the carrier of electronic circuits, the substrate’s heat resistance, chemical resistance, flexibility and other properties directly determine its applicability in the field of printed electronics. At present, in related studies at home and abroad, the applicable substrates for inkjet-printing nano conductive inks are mainly focused on photo paper, plastic (such as PET, Polyimide (PI)), and glass. These substrates have their own characteristics. Photo paper is cheap, accessible, environmentally friendly, and bendable. It has great advantages in the fields of disposable printed electronics and flexible printed electronics, especially RC high glossy photo paper which is the preferred material for inkjet printing of conductive patterns [21]. However, its high temperature resistance is poor, and the surface is prone to cracks when the temperature is higher than 120 °C. There are many types of plastic, of which PET- and PI-substrate can be used for printed electronic technology. PET substrate is cheap, but its high temperature resistance is slightly poor. If it is heated at 150 °C for a long time, irreversible deformation will occur. However, the heating temperature is lower than 150 °C in the proposed DMF fabrication technology. PI substrate, as a promising engineering plastic, can withstand a high temperature of about 500 °C, but it is expensive. Glass is a rigid substrate and cannot be bent, which is contrary to our intention to choose a flexible substrate. Therefore, we finally chose PET as flexible substrate. Due to the smooth surface structure of PET substrate, it is difficult to obtain good ink adhesion in printing, which makes the conductive ink layer deposited on the substrate uneven, resulting in poor conductivity of the driving electrode [22,23]. Therefore, the surface of PET substrate was pretreated before printing. The substrate is coated with a layer of water-based coating which is a film-forming dispersion with good leveling, great compatibility, high stability and good film-forming property, and can be used on various substrate surfaces. The substrate is baked for about 15 min at 100 °C after coating.

### 2.4. Inkjet Printing of Patterned Electrode Array

An array of patterned electrodes was designed by vector drawing software (Microsoft Visio 2016, Microsoft office, Redmond, WA, USA), and then converted into 1-bit TIF diagram. The electrode diagram was inkjet-printed by a 3D circuit board printer (BroadJET L3000, Beijing BroadTeko Intelligent Technology Co., Ltd.). The printer is equipped with 1440 industrial nozzles, the smallest ink drop is 3.5 pL, the mechanical repeatability is 0.01 mm, the maximum printing size is 297 mm × 210 mm, the printing accuracy, speed and resolution are 0.05–0.075 mm, 250 mm/s, 1440 × 1440 dpi, respectively. Inkjet printing has a sponge adsorption effect, so the printed lines are often imprinted, especially for the conductive ink, which affects the conductivity of the lines [22]. The usual solution is a multi-layer printing mode to get sufficient conductivity. We printed the electrode diagram with different layers to study the effect of printing of silver traces on conductivity. For each printing, it was dried before printing the next time. After the last printing, the conductive ink on the substrate was sintered at 120° for 30 min to ensure the solidification of silver electrodes and reduce the resistance [24,25]. We used a scanning electron microscope (SEM) (Quanta 250, FEI, Hillsboro, OR, USA), atomic force microscope (AFM) (Dimension FastScan, Bruker, Karlsruhe, Germany) and digital multimeter to characterize the printed electrodes. Furthermore, the PET substrate is fixed onto a glass slide with double-sided adhesive tape after printing the electrodes to ensure the flatness of the FDMFC.

### 2.5. Preparation of Food Wrap Film as the Dielectric Layer

At present, there are three types of food wrap films commonly used in the market, namely polyethylene (PE), polyvinylidene chloride (PVDC) and polymethylpentene (PMP). Teflon^®^ AF, as the hydrophobic layer, is coated on the food wrap film and heated at high temperature to remove all solvent, which can give a smoother coating and improves the polymer’s adherence to the surface. The heat resistance temperature of PE, PVDC and PMP wrap films are 110 °C, 140 °C and 180 °C, respectively. Therefore, we chose PVDC and PMP wrap films as the dielectric layer. The thickness of the four brands of PVDC films we purchased are all about 12 μm, while that of PMP film is slightly thinner, about 10.5 μm. To ensure a close attachment between the dielectric film and the electrode array without air bubbles, dielectric film is tightened and adhered to the glass slide with double-sided tape until there are no wrinkles, and a thin layer of silicone oil coats the electrode array and PET substrate before the dielectric film is adhered to the glass slide.

### 2.6. Coating of Hydrophobic Layer

Taking into account the characteristics of dielectric wrap films, spray-coating is used instead of spin-coating to coat a layer of Teflon^®^ AF on the dielectric films. Place the dielectric film vertically, spray Teflon^®^ AF evenly from up to down and from left to right with a common spray bottle, wait for a few minutes to allow excess Teflon^®^ AF to flow away along its surface and heat to form a hydrophobic film. The contact angle of the droplet on PVDC and PMP wrap film coated with Teflon^®^ AF was measured by the free software Image J through the droplet images collected by a CCD camera (HV315UC, Daheng, Beijing, China).

## 3. Results and Discussions

### 3.1. Performance of the Electrodes and Silver Traces Inkjet-Printed on PET Substrate

**Figure 2:**
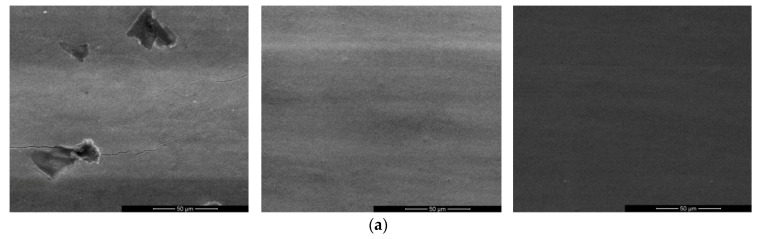

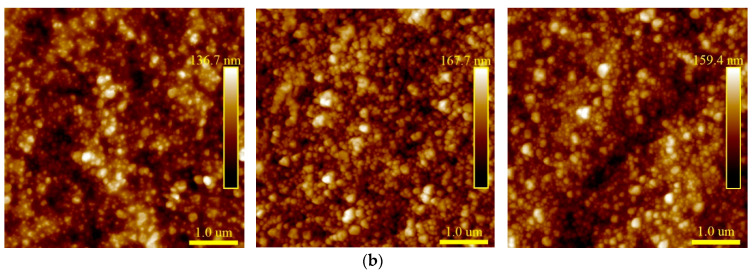
Silver electrode printed on PET substrate for different numbers of inkjet printing. (**a**,**b**) scanning electron microscope (SEM) and atomic force microscope (AFM) images of electrode at one, two and three printing.

Electrical conductivity and resolution are critical to the performance of inkjet printing FDMFC. High-quality conductivity and resolution can effectively avoid chip failure. Based on the characteristics of inkjet printing, silver traces with a small width will increase the probability of electrical disconnection and small gaps between electrodes may easily overlap with each other. However, a large gap will increase the difficulty for the droplet to cross the adjacent electrode. The 3D circuit board printer used here has two printing modes: large ink drop and small ink drop. Comparing the two different printing modes, it is found that the conductivity of silver traces printed by small ink drops and the gap between the traces significantly outperform those by large ink drops. After the small ink droplets are dried and solidified, the ink dots are smaller, and the coverage between ink droplets is more uniform, which effectively improves the conductivity; the diffusion of small ink drops on the PET substrate is relatively lower. When printing parallel silver traces, the small ink dots scattered between the traces reduce the connection probability of the traces. Therefore, the small ink drop printing mode is more suitable for FDMFC in terms of electrical conductivity and resolution. The quality of the patterned electrodes by inkjet printing with small ink drop was evaluated by SEM and AFM images in Figure 2. The images show that cracks and small pits occur after sintering in single printing, which makes the silver electrode layer discontinous, and there are some irregular scattered particles of ~50 μm dia. We speculated that the particles are silver-salt precipitates formed during the sintering process, inevitably resulting in the surface of the electrode layer being uneven; however, after multi-layer printing, there are no cracks, pits or scattered silver particles, forming better surface uniformity of the silver electrode layer. Therefore, the multi-layer printing mode can effectively avoid the surface defects of the silver electrode layer in the single printing. AFM images are used to assess the surface roughness of silver electrode layer. The multi-layer printing mode reduces the surface roughness (*R*a) to about 20 nm.

**Figure 3:**
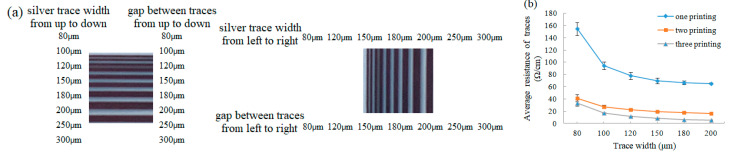
Characterization of resolution and conductivity of the inkjet-printed FDMFCs. (**a**) Photo of printed silver traces showing gradients of trace widths and gap between traces (from 80 to 300 μm) in horizontal and vertical directions. (**b**) average resistance of silver traces as a function of trace width for different numbers of inkjet printing on PET substrate.

The output spatial resolution and conductivity of the electrodes and silver traces can be observed in Figure 3. It shows the contrast and clarity of silver traces with different width and electrodes with different gaps. When the gap between silver traces is small, there are small scattered ink droplets. It is not until the gap increases to 150 μm that the gap in both horizontal and vertical directions can be clearly distinguished. The actual width is 40% larger and the actual gap is 40% smaller than the design value. Figure 3b demonstrates the resistance of silver traces with different width for different printing numbers. With the increase in trace width (from 80 μm to 300 μm), the resistance of the silver trace gradually decreases and the silver trace with the larger width shows better continuity and conductivity. Moreover, regardless of trace width, the average resistance of silver traces decreases with the increase in number of prints. The resistance was greatly reduced for two printings and was slightly lower for three printings. When printing once, since less silver is deposited on the substrate, the silver film is not uniform and the silver traces may be broken, resulting in high resistance. The main reason for the sharp decrease in trace resistance for the two printings is that the second printing constitutes a multi-layer printing mode, depositing more silver, which improves the compactness of the microstructure of traces and strengthens the connectivity between silver particles to form a continuous conductive path. When the number of prints continues to increase, although the increase in silver helps the formation of clear and complete silver traces, the amount of resin in the ink also increases. Too much resin can affect the contact between the conductive fillers, so there is no significant reduction in average resistance for the third printing. The average resistance of the silver traces with 80 μm width processed by photolithography is about 40.19 Ω while that of two and three printing are 41.38 Ω and 33.54 Ω, respectively, which shows that the conductivity in multilayer printing mode is comparable to that in photolithography technology. Although the increase in printing number can improve the conductivity, it will reduce the output spatial resolution, which increases the probability of overlap between electrodes in the printed pattern of FDMFCs, thereby affecting the performance of droplet operations and resulting in misoperation. Therefore, the three printing mode is used in all the following FDMFC experiments. The thickness of the silver trace in this printing mode is about 110 μm.

### 3.2. The Properties of Dielectric Film and Hydrophobic Layer

**Figure 4:**
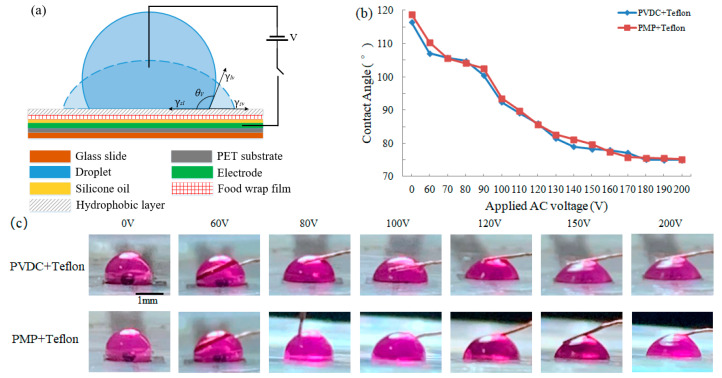
(**a**) electrowetting-on-dielectric (EWOD) device, (**b**) the contact angle of 3 μL potassium permanganate droplet on polyvinylidene chloride (PVDC) and polymethylpentene (PMP) wrap film coated Teflon^®^ AF 1600 versus the applied AC voltages at 400 Hz, (**c**) the spreading photos of 3 μL potassium permanganate droplet on PVDC and PMP wrap film coated Teflon^®^ AF 1600 under the applied AC voltage.

**Figure 5:**
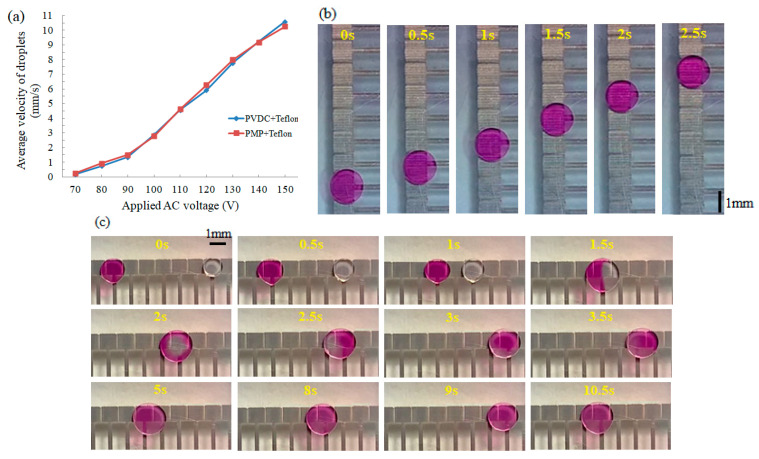
Demonstration of droplet operations on the square electrode array of FDMFC. (**a**) the velocity of droplet on PVDC- and PMP-based FDMFCs. (**b**,**c**) Series of video frames displaying transport, merging and mixing of droplets on a liner square electrode array of PVDC-based FDMFC under the AC voltage of 100 V_rms_ at 400 Hz.

For a droplet on the printed electrode with dielectric film and Teflon^®^ AF, the contact angle (CA) was measured as a function of the applied voltage by the EWOD device (Figure 4a) to explore the dielectric properties of PVDC and PMP wrap film coated Teflon^®^ AF. The spreading images of the droplet was collected by a digital camera and processed by ImageJ software. As shown in Figure 4b,c, the initial CA under no applied voltage is close to 120 °C, which is consistent with the results in previous literatures [26,27,28]. The change trend of CA of the droplet on two dielectric films versus the applied AC voltage at 400 Hz is very similar and both of CAs reach saturation when the applied voltage is higher than 180 V_rms_. Through droplet movement experiments, it was found that for the printed chips of two dielectric films, the droplet started to move only when the applied voltage reached 70 V_rms_, but it is extremely slow; it is not until the applied voltage increases to 90 V_rms_ that the droplet can move smoothly, as shown in Figure 5a. Such a voltage is higher than that required on the chips fabricated by the traditional photolithography technology. It is due to the excessive thickness of PVDC and PMP wrap film (both greater than 10 μm) that a higher voltage is required to drive the droplets. Under the same voltage, the velocity of droplets on two kinds of FDMFC is equivalent. Therefore, the two kinds of food wrap films are suitable for FDMFC in term of CA and the velocity of the droplet.

### 3.3. Droplet Operations on FDMFC

According to Figure 1a,c, we fabricated FDMFCs economically and easily. The single FDMFC costs approximately $3, which includes material and technician time, and is much lower than that fabricated by traditional photolithography technology. Such a low cost and simple technology enable users to get rid of the dependence on expensive photolithography technology and related facilities, which is the first advantage of DMF fabrication technology proposed in this paper. Moreover, it takes only one hour to fabricate a chip by the proposed technology. In addition, the FDMFC is not disposable, but can be used repeatedly. If necessary, just replace the dielectric wrap film coated with hydrophobic layer, and the printed FDMFC can continue to be used. This is far different from the traditional dielectric coating method in which the dielectric material (such as Parylene-C) is attached to the electrodes. This feature is the second advantage of the new DMF fabrication technology. The FDMCs processed by this technology can realize stable and smooth droplet movement. Droplet operations can be performed successfully on square and hexagon electrode arrays on PVDC- and PMP-based FDMFC.

#### 3.3.1. Droplet Operations on Square Electrodes

Figure 5a shows the velocity of a droplet on PVDC- and PMP-based FDMFC. The velocity of the droplets on two chips is similar, which indicates that the performances of PVDC- and PMP-based FDMFC are equivalent. With the AC voltage of 100 V_rms_ at 400 Hz, a 1 μL droplet was actuated along square electrode array on a PVDC-based FDMFC, as shown in Figure 5b (Video S1). One of the main functions of DMF chips is to be used for biochemical analysis. Mixing plays an important role in biochemical analysis because the time required for mixing is dominant [29]. Figure 5c demonstrates a complete droplet mixing operation at the same AC voltage. Two droplets of different sizes were simultaneously actuated to move along their respective paths and merge at the intermediate electrode. The merged droplet moved back and forth on the liner electrode array mixer to accelerate the mixing process until it was completely mixed (Video S2). In the whole process of droplet operation, even when the AV voltage is increased to 300 V_rms_, there is no dielectric breakdown, which shows that the problem of dielectric breakdown is not prone to happen on this kind of DMF chip.

#### 3.3.2. Droplet Operations on Hexagon Electrodes

**Figure 6:**
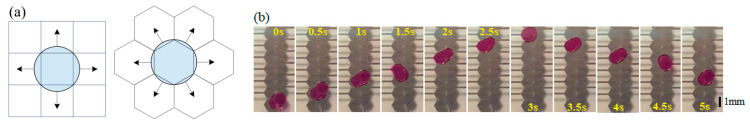
Demonstration of droplet operations on the hexagon electrode array of FDMFC. (**a**) direction of the droplet movement on square and hexagon electrode array. (**b**) the movement of the droplet in zigzag mode or along parallel direction on the hexagon electrode array at the AC voltage of 100 V_rms_.

The hexagon is more similar to the droplet shape in the closed DMF than the square, so the hexagon electrode array can offer many advantages over the square electrode array in droplet movement, mixing and splitting operations and velocity, etc. [30,31,32]. For example, the movement of the droplet on the former is more flexible than that on the latter, since the droplet theoretically has six movement directions on the former while it only has four movement directions on the latter, as shown in Figure 6a. Figure 6b demonstrates that the droplet moved freely in zigzag mode or along parallel direction on the hexagon electrode array at the AC voltage of 100 V_rms_ (Video S3).

## 4. Conclusions

We introduced a simplified technology for the fabrication of DMF in which the electrodes were generated by inkjet printing nanosilver conductive ink on the flexible PET substrate, PVDC− and PMP−food wrap film was used as the dielectric layer and Teflon^®^ AF was sprayed to form a hydrophobic layer. As long as the patterned electrodes can be drawn by drawing software, and within the resolution range of the printer, they can be printed out by inkjet printing. Therefore, the inkjet printing method facilitates electrodes with variable and complicate shapes which undoubtedly extends the application range of DMF. The replacement of traditional dielectric material with food wrap film greatly increases the utilization rate of DMF chips and further reduces the fabrication and use costs. In addition, spray-coating Teflon^®^ AF with a common spray bottle can break away from the dependence on expensive spin-coating facilities. The proposed design and fabrication technology of FDMFCs is superior to the reported rapid prototyping technology of DMF in term of cost, time, material selection, chip utilization and production capacity, which is more accessible to general users of DMF. FDMFCs was demonstrated to perform various droplet operations on the electrode arrays with different shapes by EWOD, such as transport, merging and mixing. We infer that the FDMFCs design and fabrication technology will become an important development direction in the future and act as a promising alternative to the traditional technology.

## Supplementary Materials

The following are available online at https://www.mdpi.com/2072-666X/11/12/1113/s1, Video S1: Droplet transport on the square electrode array, Video S2: Merging and mixing of two droplets on the square electrode array, Video S3: Droplet transport on the hexagon electrode array.
